# Genetic fusion of P450 BM3 and formate dehydrogenase towards self-sufficient biocatalysts with enhanced activity

**DOI:** 10.1038/s41598-021-00957-5

**Published:** 2021-11-04

**Authors:** Arsenij Kokorin, Pavel D. Parshin, Patrick J. Bakkes, Anastasia A. Pometun, Vladimir I. Tishkov, Vlada B. Urlacher

**Affiliations:** 1 Institute of Biochemistry, Heinrich Heine University Düsseldorf, Universitätsstr. 1, 40225 Düsseldorf, Germany; 2 Department of Chemistry, Lomonosov Moscow State University, 119991 Moscow, Russian Federation; 3 Laboratory of Molecular Engineering, Federal Research Centre “Fundamentals of Biotechnology” RAS, 119071 Moscow, Russian Federation; 4 Present Address: Institute of Bio- and Geosciences, IBG-1: Biotechnology, Forschungszentrum Jülich GmbH, 52425 Jülich, Germany

**Keywords:** Biotechnology, Molecular engineering, Biochemistry, Enzymes

## Abstract

Fusion of multiple enzymes to multifunctional constructs has been recognized as a viable strategy to improve enzymatic properties at various levels such as stability, activity and handling. In this study, the genes coding for cytochrome P450 BM3 from *B. megaterium* and formate dehydrogenase from *Pseudomonas* sp. were fused to enable both substrate oxidation catalyzed by P450 BM3 and continuous cofactor regeneration by formate dehydrogenase within one construct. The order of the genes in the fusion as well as the linkers that bridge the enzymes were varied. The resulting constructs were compared to individual enzymes regarding substrate conversion, stability and kinetic parameters to examine whether fusion led to any substantial improvements of enzymatic properties. Most noticeably, an activity increase of up to threefold was observed for the fusion constructs with various substrates which were partly attributed to the increased diflavin reductase activity of the P450 BM3. We suggest that P450 BM3 undergoes conformational changes upon fusion which resulted in altered properties, however, no NADPH channeling was detected for the fusion constructs.

## Introduction

In times of an emerging importance of the bio-based, sustainable economy, biocatalysis has gained particular attention as a promising way to complement chemical synthesis by utilizing regio- and stereoselective enzymes. Application of enzymes on an industrial scale has been established for several processes and the field is in continuous development^1,2^. In this respect, optimization of the reaction systems that involve more than one enzyme, have attracted much attention in the recent decade^3,4^. Significant efforts in this field of research deal with improving enzymatic properties through variation of the microenvironment of enzymes rather than altering enzymes’ structures by protein engineering. Strategies such as enzyme (co)-immobilization, co-assembly of enzymes to higher ordered complexes and enzyme fusion have been successfully applied for various reactions in vivo and in vitro as recently reviewed^5^. Enzyme fusion affects proximity of active sites by linking multiple individual enzymes to a single unit. The process can be conducted either post translationally or through the more prominent approach of creating one gene sequence out of the several individual gene sequences. Spatial distance between two enzymes in fusion can be modulated by the introduction of peptide linkers^6,7^. They can vary in length, rigidity and stability according to their amino acid composition^8^. Order of the fused genes (N- and C-terminal orientation) and the choice of linker have been reported to influence correct folding of the fusion constructs, their oligomerization state, stability, and activity^7,9–15^. Common for the fused systems is that the active sites of enzymes are located in close proximity to each other and thereby substrate channeling could occur. This effect describes a direct passing of the product of one enzyme reaction as an intermediate to the second reaction without equilibration with the bulk solvent^16^. In a cellular environment, it is proposed to act as a control mechanism at branch points in metabolic pathways and as a protection mechanism from labile/toxic intermediates^17,18^. A positive effect of enzyme proximity has been reported also in in vitro applications^5^, although substrate channeling in these cases remains highly debated^19–21^.

Under consideration of these criteria, enzymes from various classes have been merged to bifunctional units that exhibit improved properties compared to their individual enzyme parts. For cascade reactions, fusions were created for example between alcohol dehydrogenases (ADH) and Baeyer–Villiger monooxygenases (BMVO) that led to an improved substrate conversion in in vitro and in vivo reactions alike^9,10,22^. Similar results were achieved for fusions between an ADH and an aminotransferase^13^, and an aldolase and a kinase^23^. Moreover, dehydrogenases were also fused to enzymes that are dependent on costly nicotinamide cofactors to introduce a regeneration system. Thereby, fusions of ADHs,formate dehydrogenases (FDHs) and phosphite dehydrogenase (PTDH) to other dehydrogenase, BMVO’s as well as cytochrome P450 monooxygenases (CYP or P450) were reported^24–26^.

Cytochrome P450 BM3 (CYP102A1) is a fatty acid hydroxylase that consists of a FAD- and FMN-containing reductase domain (BMR) and the heme-containing monooxygenase domain (BMP). High efficiency of both electron delivery from NADPH to the heme group, mediated by BMR, and subsequent substrate oxidation catalyzed by BMP, can be highlighted by arachidonic acid hydroxylation with a k_*cat*_ of 17,100 min^−1^, the highest reported value for P450s yet^27^. Numerous mutants of P450 BM3 were created enabling the synthesis of valuable compounds ranging from pharmaceutical metabolites to aroma compounds and fine chemicals. However, dependency on the cofactor NADPH makes large-scale applications of P450 BM3 expensive when the cofactor is used in stoichiometric amounts. To circumvent this issue, additional enzymes such as glucose dehydrogenase (GDH)^28^, PTDH^29^ as well as FDH^30^ have been employed for in situ cofactor regeneration by turnover of cheap sacrificial substrates. In this regard, FDH is appealing as it catalyzes the reduction of NAD(P)^+^ to NAD(P)H upon formate oxidation to gaseous CO_2_^31^. FDH from *Pseudomonas *sp. 101 with high thermal and solvent stability has often been applied for cofactor regeneration^32,33^. While the native FDH from *Pseudomonas *sp. 101 prefers NAD^+^, site-directed mutagenesis resulted in variants with a shifted nicotinamide cofactor specificity towards NADP^+^ that were successfully applied in cofactor regeneration experiments with P450 BM3 before^31,34,35^.

The goal of this work was to create and characterize enzyme fusions between P450 BM3 and a NADP^+^-specific variant of FDH from *Pseudomonas *sp. 101, in comparison to the system comprised of the individual enzymes. We investigated the influence of enzyme order in the fusion constructs and the effects of a rigid proline linker and a flexible glycine linker of various length between the fused partners. Kinetic parameters, thermal and solvent stability were determined for the fusions and compared to the individual enzymes, and probability for substrate channeling in fusions was investigated.

## Results

### Design, expression and quantification of fusions

**Figure 1:**
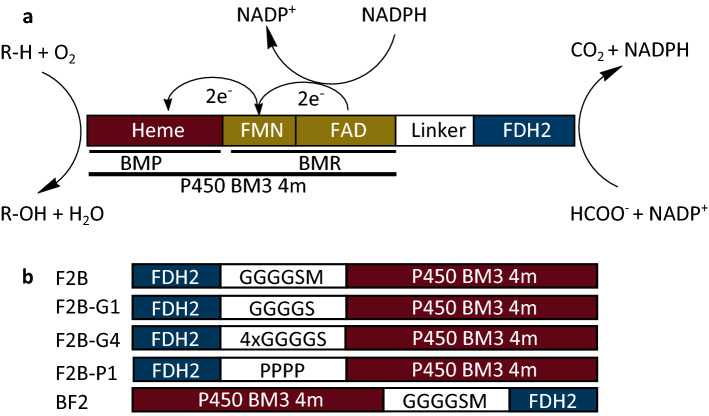
Design of enzyme fusions of BM3 4m and FDH2. (**a**) Detailed architecture and reaction scheme of the P450 BM3-FDH fusions as exemplified by the fusion construct with the enzyme order BF2 (real sizes of the enzymes are not considered). The formate dehydrogenase FDH2 (blue colored) catalyzes the oxidation of HCOO^−^ to CO_2_ and reduction of NADP^+^ to NADPH. Cytochrome P450 consists of a BMP and BMR domain. The BMR domain (greenish colored) contains an FAD cofactor which partakes in the oxidation of NADPH to NADP^+^ and transfers two electrons to FMN, and an FMN cofactor which transfers the electrons (2 × e^−^) to the BMP domain (red colored). Subsequent substrate hydroxylation occurs at the active site of the BMP domain which contains heme and reduces O_2_ to water upon substrate hydroxylation. In the fusion enzyme, BM3 4m and FDH2 are bridged by a linker (white colored). (**b**) Schematic diagram for the constructed fusions with FDH2 (blue colored) positioned at the N-terminal and BM3 4m (red colored) positioned at the C-terminal (enzyme order F2B) and vice versa (enzyme order BF2). Short amino acid linkers are illustrated through the white box with the noted amino acids describing the composition of the linkers. The fusion constructs F2B and BF2 contain the methionine of the enzyme at the C-terminal position which was removed in the fusion constructs F2B-G1, F2B-G4 and F2B-P1.

The P450 BM3 mutant A74G/F87V/L188G/R471C (further referred to as BM3 4m) with activity towards fatty acids and several non-physiological substrates like the sesquiterpenoid β-ionone was used in our study. As a partner for fusions, we chose FDH from *Pseudomonas *sp. 101 (referred to as FDH2) with the mutation D221Q that induces NADP^+^ specificity matching the cofactor preference of P450 BM3^31^. With the process of enzyme fusion, substrate oxidation by BM3 4m and NADPH regeneration by FDH2 would be combined in one construct (Fig. 1a). In the designed fusions, either the N-terminal FDH2 and C-terminal BM3 4m (F2B) or the N-terminal BM3 4m and C-terminal FDH2 (BF2) were bridged by a flexible glycine linker (GGGGS). Since impaired FDH activity was reported by other groups when fusion enzymes harbored a formate dehydrogenase at the C-terminal part^36,37^, along with the fusion with the short glycine linker F2B-G1 (GGGGS) further fusions were constructed in the FDH2-BM3 4m order with a proline linker F2B-P1 (EPPPPK) and a long glycine linker F2B-G4 (GGGGS)_4_ (Fig. 1b, Table 1). To exclude any possible effects during transcription and protein expression, the start codon of the C-terminal fusion partner BM3 4m was deleted in the constructs F2B-P1, F2B-G4 and additionally in F2B leading to the F2B-G1 construct. All constructs harbored an N-terminal His-tag to facilitate the subsequent purification.

**Table 1:** Fusion constructs consisting of BM3 4m and formate dehydrogenase FDH2. Constructs that lack the ATG start codon are marked as (–ATG). The linkers are displayed as the corresponding amino acid sequences. The quantification of expression levels was conducted by recording CO-difference spectra with cell lysates. Subsequently, the concentration was normalized to cell wet weight. Each value is depicted as the mean of triplicate measurement and error bars represent the standard deviation.

Fusion protein	N-terminal enzyme	C-terminal enzyme	Linker sequence	(mg) P450 g^−1^_cww_
BF2	BM3 4m	FDH2	SGGGGS	24.0 ± 1.1
F2B	FDH2	BM3 4m	SGGGGS	7.9 ± 0.5
F2B-P1	FDH2	(–ATG) BM3 4m	EPPPPK	6.6 ± 0.5
F2B-G1	FDH2	(–ATG) BM3 4m	SGGGGS	7.6 ± 0.5
F2B-G4	FDH2	(–ATG) BM3 4m	SGGGGS × 4	7.0 ± 0.2

All fusion constructs as well as individual BM3 4m and FDH2 were expressed in *E. coli* BL21 (DE3) and the heme-containing enzymes were quantified in the CO difference spectrum assay (Table 1). We observed that enzyme order influenced the concentration of the expressed fusion constructs considerably. The BF2 order resulted in a higher expression level (24 mg g_cww_^−1^) than F2B (7–8 mg g_cww_^−1^) and even surpassed quantities of the individually expressed BM3 4m (19 mg g_cww_^−1^) (Table 1). The expression levels among F2B-P1, F2B-G1 and F2B-G4 did not differ significantly, indicating that neither linker rigidity nor linker length had any observable impact on expression. Further analysis by SDS-PAGE revealed that the bands of the fusion enzymes were clearly distinguishable (circa 160 kDa) from the bands of BM3 4m (circa 120 kDa, calculated Mw 117 kDa) and FDH2 (circa 45 kDa, calculated Mw 44 kDa) and fit the calculated molecular weight of 163 kDa (Supplementary Fig. S1). Individual enzymes and fusion constructs were subsequently purified by immobilized metal affinity chromatography (IMAC) and size exclusion chromatography (SEC). Spectral analysis revealed no significant deviations between individual BM3 4m and the fusion constructs F2B-G1 and BF2 (Supplementary Fig. S2). All three enzymes exhibited an intensive Soret band at 420 nm as well as indications of the α/β bands at 570/535 nm, both of which are characteristic for the heme group^38^. Thus, the effects of enzyme fusion appear to be marginal on the coordination of the prosthetic heme group. However, partial degradation of the fusion constructs occurred after purification and increased over time in a specific manner with an additional fragment at 45 kDa for F2B fusion and at 120 kDa for BF2 fusion. The BF2 fragments showed an absorbance at 420 nm during SEC (Supplementary Fig. S3), while the isolated fragment of F2B exhibited FDH activity*.* Judging from these results and the molecular weights of FDH2 and BM3 4m, these fragments presumably originate from the separate expression or cleavage of the fusion partners after expression or during purification. This kind of degradation enhanced over time and occurred in the presence of the serine protease inhibitor phenylmethylsulfonyl fluoride (PMSF). The investigation of the constructs F2B-G1, F2B-P1 and F2B-G4 without the start codon in the C-terminal fusion partner, that might act as an alternative open reading frame, revealed the same protein bands’ pattern after expression and purification (Supplementary Fig. S1)^39^. Obviously, neither different linkers nor the deleted start codon prevented the appearance of separate fragments. Even though PMSF as specific inhibitor of serine proteases did not prevent degradation, proteolytic cleavage mediated by another type of proteinases cannot be excluded. A protease cleavage site was reported for FDH from *Mycobacterium vaccae* N10 at the C-terminus^40^. Since FDH from *Pseudomonas *sp. 101 and *M. vaccae* N10 have the same C-terminus sequence, the observed fragmentation of the FDH2-BM3 4m fusion constructs might be a result of proteolytic cleavage^41^.

### Enzyme fusion markedly improves substrate conversion compared to individual enzymes

**Figure 2:**
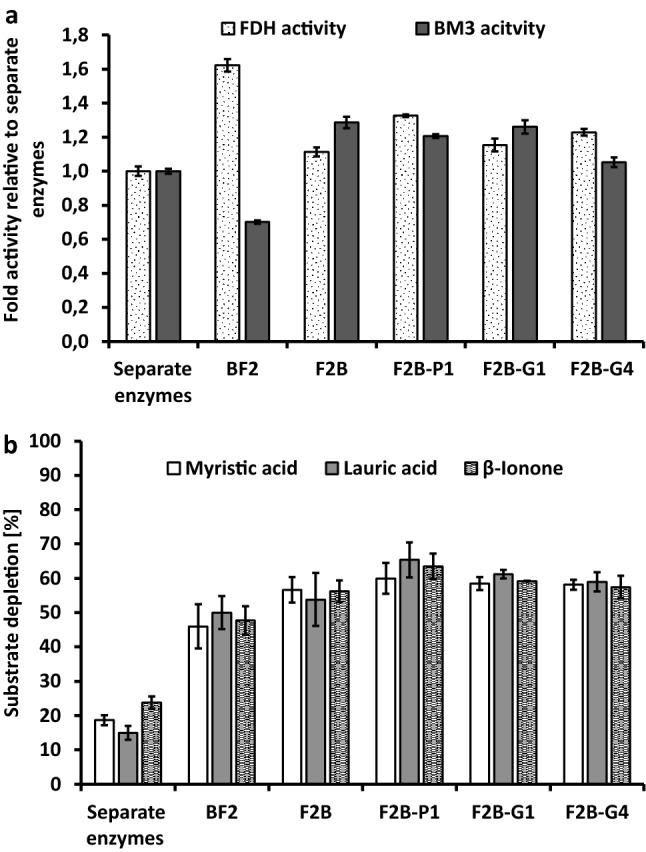
Substrate conversion by individual BM3 4m and FDH2 and their fusions. (**a**) P450 (50 nM) activity measured with 12-*p*NCA (0.2 mM, dissolved in DMSO at 2% (v/v)), NADPH (1 mM) in potassium phosphate buffer (KPi, 50 mM pH 8.1). FDH (100 nM) activity measured with sodium formate (0.6 M), NADP^+^ (0.5 mM) in KPi (50 mM, pH 7.5). (**b**) Conversions of myristic acid, lauric acid and β-ionone (0.2 mM) by P450 with concomitant NADPH regeneration via FDH-catalyzed formate oxidation: individual and fused enzymes (50 nM), NADP^+^ (0.63 mM for fatty acids, 1 mM for β-ionone), sodium formate (1.5 M) in KPi buffer (50 mM, pH 7.5). The displayed values are means of triplicate measurements with standard deviation as error bars.

We expected dual activity of the fusion constructs. To assess the integrity of both components, the FDH and the P450, their activities were tested within the fusion constructs and expressed relative to the activities of individual FDH2 and BM3 4m (Fig. 2a). Activity of FDH was quantified through NADPH generation during formate oxidation, resulting in 9 mU/mL for individual FDH2. Relative FDH activity of BF2 was 1.6-fold higher, while F2B and its variants with different linkers resulted in an 1.1- to 1.3-fold increase in FDH activity. P450 activity was examined with 12-*para*-nitrophenoxydodecanoic acid (12-*p*NCA) in the presence of NADPH, resulting in 8.3 mU/mL for the individual BM3 4m. While a decrease was observable for BF2 (0.7-fold), P450 activity of the F2B fusions with short glycine linkers and the proline linker was around 1.2- to 1.3-fold higher (Fig. 2a). Thus, both enzyme functions were detectable in the fusion constructs that were at least as effective as the individual enzymes. Remarkably, BF2 order resulted in higher FDH activity, while fusions with the fusion with F2B order showed increased activity of both fusion partners. Specifically, the highest increases in P450 activity were recorded with fusion constructs with the short glycine linker, while a proline linker was more favorable for the increase of FDH activity. Similarly, utilization of a longer glycine linker resulted in improved FDH activity but virtually no effect on P450 activity was observable.

These observations prompted further experiments on substrate conversion and GC–MS analysis to validate the positive effects mediated by enzyme fusion for in situ cofactor regeneration. Due to the natural function of P450 BM3 as fatty acid hydroxylase, the middle chain-length fatty acids lauric acid (C12) and myristic acid (C14) were chosen as substrates (Fig. 2b). Hydroxylation catalyzed by P450 BM3 4m occurs at the sub-terminal (ω-1, ω-2 and ω-3) positions^27,42^. Since BM3 4m and FDH2 are present in a 1:1 ratio in the fusions, the same ratio was used in the measurements with the individual enzymes. With the F2B fusions, conversions of up to 65% for lauric acid and 60% for myristic acid were achieved. Comparatively, reactions with BF2 fusion resulted in lower conversion of 36% for lauric acid and 43% for myristic acid. After the same reaction time, individual BM3 4m supported by the individual FDH2 at a ratio 1:1, resulted in markedly lower conversion of lauric acid (15%) and myristic acid (19%) (Fig. 2b). The product distribution patterns after the hydroxylation of lauric acid were identical for the individual and fused BM3 4m (Supplementary Fig. S4). During the conversion of myristic acid, individual BM3 4m produced 12% less ω-1 hydroxylated product compared to the fusions, thereby resulting in a slight difference in product distribution between individual and fused enzymes (Supplementary Fig. S5). Hydroxylation of the sesquiterpenoid β-ionone to 4-hydroxy-β-ionone catalyzed by BM3 4m has been reported in our previous work^43,44^. In this study, β-ionone conversion of up to 63% was achieved with the F2B fusions and 48% with BF2. Reactions catalyzed by individual BM3 4m supported by FDH2 (ratio 1:1) resulted in the lowest conversion of 24% (Fig. 2b). In conclusion, the observations illustrate more efficient in situ cofactor regeneration in the fusion constructs than by BM3 4m supported by the equimolar concentration of FDH2. The measured activity of fused enzymes was consistently two- to three-fold higher. The F2B constructs resulted in more pronounced improvements in activity than the fusion construct BF2. When comparing the different variants of F2B, the choice of linkers between the two enzymes only had marginal effects on the activity of the fusions. Herein, reactions with the construct F2B-P1 resulted in the highest values for substrate conversion. A comparison of substrate conversions achieved with fusions that harbor the short G1 and the long G4 glycine linkers also indicated that the linker length did not influence efficiency of the fusions (Fig. 2b).

Coupling efficiency is another parameter for P450 mediated reactions which determines the percentage of redox equivalents derived from NAD(P)H that lead to product formation^45^. Accordingly, the coupling efficiencies were determined for the conversion of lauric acid, myristic acid and β-ionone (Table 2). Here, the values among the individual BM3 4m and fusions were very similar and were dependent only on the substrate used. It can be presumed that enzyme fusion only had a negligible effect on coupling efficiency.

**Table 2:** Coupling efficiency of individual BM3 4m and the fusions F2B and BF2. The errors are the standard deviation.

Coupling efficiency	With lauric acid (%)	With myristic acid (%)	With β-ionone (%)
BM3 4m	78 ± 6	67 ± 10	89 ± 1
F2B	80 ± 7	74 ± 1	90 ± 3
BF2	80 ± 3	78 ± 5	84 ± 9

### Enzyme fusion induces changes in the kinetic parameters

The measured substrate conversion indicated that the enzyme fusion improves the catalytic performance relative to the unfused enzymes. To further verify these observations, kinetic parameters were determined for individual enzymes as well as the fusions F2B and BF2. The estimation of k_*cat*_ and K_M_ values for NADP^+^ reduction and HCOO^−^ oxidation was conducted with varying substrate concentrations (Table 3, Supplementary Fig. S6a,b). Individual FDH2 exhibited the lowest K_M_ values for both HCOO^−^ and NADP^+^. In comparison, the determined K_M_ values were up to 1.5-fold higher for BF2 and up to 2.2-fold higher for F2B. However, the highest k_*cat*_ values were recorded with F2B for both HCOO^−^ oxidation and NADP^+^ reduction followed by BF2 and finally individual FDH2. The kinetic parameters of individual and fused BM3 4m were determined at different 12-*p*NCA concentrations at a fixed NADPH concentration (Table 4, Supplementary Fig. S6c). The lowest K_M_^12-*p*NCA^ value was measured for the fusion construct BF2, while k_*cat*_ was the highest for F2B. Individual BM3 4m exhibited the lowest k_*cat*_ value out of the three tested enzymes. Conclusively, the observed increase in activity of the fusions was verified through the measurement of the k_*cat*_ values. However, the higher K_M_ values indicate that substrate binding was impacted for fused FDH2 and BM3 4m at the fusion order F2B. Thus, the BF2 enzyme order had a lesser influence on K_M_ values than F2B but also resulted in lower k_*cat*_ values. Irrespective of the increases in K_M_, catalytic efficiency was higher for the fusion enzymes compared to individual FDH2 and BM3 4m.

**Table 3:** Kinetic parameters measured for individual FDH2 and fusions during NADP^+^-dependent HCOO^-^ oxidation and HCOO^−^-dependent NADP^+^ reduction. The kinetic parameters with the appropriate standard errors were calculated using the software *Origin 9* and fitted to the *Michaelis–Menten* model.

NADP^+^ reduction	Apparent ${\text{K}}_{\text{M}}{^{\text {NADP}^+}}$ (mM)	Apparent k_*cat*_ (min^−1^)	k_*cat*_/K_M_ (mM^−1^ min^−1^)	HCOO^−^ oxidation	Apparent ${\text{K}}_{\text{M}}{^{\text {HCOO}^-}}$ (mM)	Apparent k_*cat*_ (min^−1^)	k_*cat*_/K_M_ (mM^−1^ min^−1^)
FDH2	0.17 ± 0.01	100 ± 1.5	595	FDH2	36.4 ± 5.0	80 ± 2.6	2.2
F2B	0.4 ± 0.06	256 ± 14.1	640	F2B	60 ± 5.5	200 ± 5.2	3.3
BF2	0.26 ± 0.03	177 ± 5.6	680	BF2	38.5 ± 1.3	159 ± 1.5	4.1

**Table 4:** Kinetic parameters of individual BM3 4m and fusions determined during NADPH-dependent 12-*p*NCA hydroxylation. The kinetic parameters with the appropriate standard errors were calculated using the software *Origin 9* and fitted to the *Michaelis–Menten* model.

12-*p*NCA hydoxylation	Apparent K_M_^12-*p*NCA^ (µM)	Apparent k_*cat*_ (min^−1^)	k_*cat*_/K_M_ (µM^−1^ min^−1^)
BM3 4m	21 ± 2.3	58.7 ± 6.6	2.8
F2B	25.9 ± 2.7	210 ± 22.3	8.1
BF2	17.4 ± 1.6	75.2 ± 7.1	4.3

### Activity of the reductase domain BMR in fusions

As the fusions of BM3 4m and FDH2 demonstrated higher activities and k_*cat*_, we attempted to identify the origin of the enhancement. Activity of the reductase domain BMR within the fusions was monitored using artificial electron acceptors. To this end, ferricyanide reduction by the FAD-domain and cytochrome c reduction by the FMN-domain were determined and compared for F2B, BF2 and individual BM3 4m. We observed an increased rate of ferricyanide reduction by F2B (113 s^−1^) compared to BF2 (75 s^−1^ ± 0.6) and BM3 4m (55 s^−1^ ± 1.5). The cytochrome c reduction rate was also higher in F2B (67 s^−1^) compared to BF2 (23 s^−1^) and BM3 4m (20 s^−1^). It can be therefore concluded that reduction of FAD was increased by enzyme fusion, especially at the F2B enzyme order. It has been reported that P450 BM3 is active as dimer, and the intermolecular electron transfer occurs between the reductase domain BMR of one P450 BM3 molecule to the heme-domain BMP of the second P450 BM3 molecule within a dimer^46^. Since in F2B the FAD-binding domain is located at the C-terminus of the protein as in the unfused P450 BM3, the improved reductase activity might be due to particular oligomerization of F2B, favorable for intermolecular electron transfer. This aspect requires a more detailed investigation in the future.

### Thermal and solvent stability of fusions

**Figure 3:**
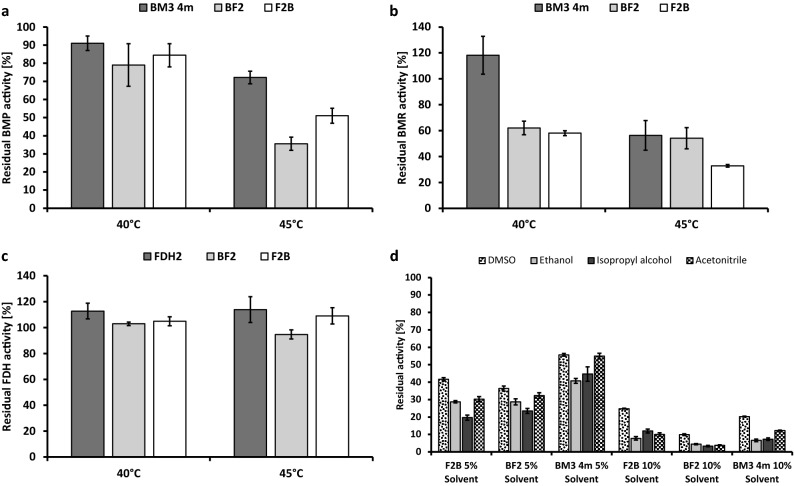
Thermal (**a**–**c**) and solvent stability (**d**) of individual BM3 4m and FDH as well as fusion constructs F2B and BF2. (**a**) Activity values were measured after incubation at 40 °C and 45 °C. The resulting values were normalized to values obtained after incubation at 25 °C (= 100%). Standard deviation is shown as error bars. Measurements were conducted in KPi buffer (50 mM, pH 8.1) with 12-*p*NCA (0.07 mM, dissolved in DMSO), NADPH (0.5 mM) and purified enzyme (50 nM). (**b**) Samples consisted of Tris–HCl (50 mM pH 7.5), potassium ferricyanide (1 mM), NADPH (0.5 mM) and purified enzyme (5 nM). (**c**) FDH activity was determined with samples consisting of KPi (50 mM, pH 7.5), sodium formate (0.6 M), NADP^+^ (1 mM) and purified enzymes (50 nM). (**d**) For the determination of solvent stability, conversion of 12-*p*NCA at 5% and 10% solvent concentration was normalized to the values measured at 2% of the appropriate solvent (= 100%). Samples consisted of KPi (50 mM pH 8.1), 12-*p*NCA (0.07 mM), NADPH (0.5 mM) and varying concentrations of DMSO, ethanol, isopropyl alcohol and acetonitrile.

Further experiments were conducted to investigate how fusion of BM3 4m and FDH2 affected their thermal and solvent stability. Previous studies have revealed that BMR has a melting temperature of around 48 °C, while BMP was more thermostable with a melting temperature of 63 °C^47^. By measuring the FMN and FAD release by heat denaturation, we observed a very similar melting temperature for BMR domain of BM3 4m (49 °C) as for F2B (50 °C) and BF2 (47 °C)^48^. Thermal stability was further analyzed by incubation of individual enzymes and fusions for 10 min at 40 °C and 45 °C with subsequent measurements of 12-*p*NCA turnover. The stability of the FAD-containing domain of BMR and FDH2 were further examined through ferricyanide and NADP^+^ reduction, respectively. No significant differences in FDH activity in the fusion constructs and individual FDH2 was observed after incubation at the appropriate temperatures (Fig. 3c). These observations coincide with previous findings on a high thermal stability for WT FDH at T_M_ 67.6 °C which was not reached during our experiments^49^. After measuring the 12-*p*NCA turnover, a higher residual activity was recorded for individual BM3 4m compared to F2B and BF2 after incubation at 45 °C (Fig. 3a). A similar trend was observed for ferricyanide reduction (catalyzed by BMR) after incubation at higher temperatures (Fig. 3b). Thus, by fusing BM3 4m and FDH2, mainly thermal stability of the reductase (BMR) component of P450 BM3 4m was negatively affected. As proposed in other work, BMR undergoes irreversible inactivation at elevated temperatures presumably through conformational changes and subsequent loss of FMN and FAD^50^.

To determine solvent stability, 12-*p*NCA hydroxylation was assessed in the presence of 5% and 10% (v/v) of either DMSO, ethanol, isopropyl alcohol or acetonitrile, and activity was normalized to the corresponding measurements in the presence of 2% solvent concentration which was added for substrate solubilization. At 5% solvent concentration, individual BM3 4m displayed residual activities ranging from 44% with ethanol to 55% with DMSO. The range of residual activity measured with F2B and BF2 was lower (Fig. 3d). In the presence of 10% organic solvents, residual activity dropped noticeably for individual BM3 4m and the fusion constructs with ethanol, isopropyl alcohol, and acetonitrile to around 10%. Thus, DMSO had the lowest impact on enzyme stability, followed by acetonitrile, isopropyl alcohol and ethanol.

### Substrate conversion in the presence of a competitive reaction

**Figure 4:**
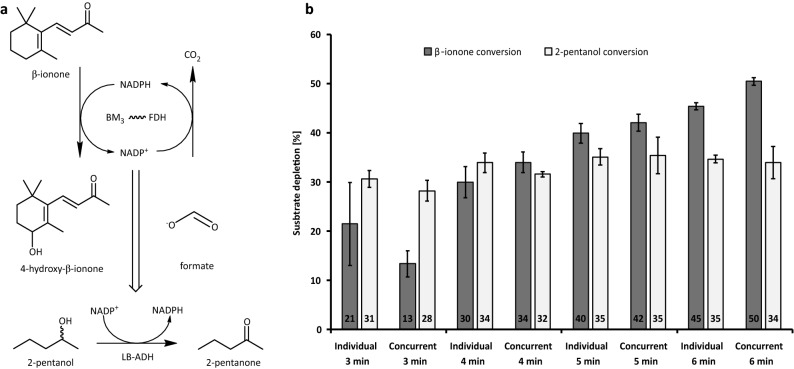
Determination of NADP^+^ channeling within the fusion construct F2B in the presence of LB-ADH as a competitive enzyme. (**a**) Reaction scheme for conversion of β-ionone to 4-hydroxy-β-ionone by a BM3-FDH fusion and the introduction of LB-ADH that competes with the FDH for NADP^+^. (**b**) Conversion of 2-pentanol to 2-pentanone by LB-ADH (ADH) and the conversion of β-ionone to 4-hydroxy-β-ionone by F2B was conducted either separately (marked as “Individual”) or in one pot (marked as “Concurrent”). Concentrations of the products were measured after the depicted time intervals.

An earlier study on kinase-aldolase fusions indicated that the introduction of a competitive reaction can be used to elucidate substrate channeling in the fusion^23^. Therein, the product of the first reaction is more likely utilized by the second enzyme located in close proximity rather than by an externally added competitive enzyme. To apply this approach to our system, conversion of β-ionone by F2B was conducted in the presence of a second enzymatic reaction. To this end, the NADP^+^-dependent (*R*)-specific alcohol dehydrogenase from *L. brevis* (LB-ADH) was added. This enzyme catalyzes the oxidation of 2-pentanol to 2-pentanone with the concomitant reduction of NADP^+^ to NADPH (Fig. 4a)^51^. When β-ionone is converted by the fusion, NADP^+^ is produced which can be used by both FDH from the fusion and by the externally added LB-ADH if 2-pentanol is present. In case of a channeling effect within the fusion construct, equilibration of NADP^+^ with the bulk solvent does not occur. In these experiments, NADPH was added for reaction initiation and oxidized during the conversion of β-ionone to gradually yield NADP^+^ at low concentrations. A 30-fold excess of LB-ADH was added to ensure that even low bulk NADP^+^ concentrations would be detected. An increasing β-ionone depletion from 21% after 3 min to 45% after 6 min was observed with only fusion F2B (Fig. 4b). Comparatively, β-ionone conversion by F2B in the presence of LB-ADH and 2-pentanol resulted in only 13% after 3 min, and 50% after 6 min. No significant difference in 2-pentanol depletion by LB-ADH was observed in the presence or absence of F2B and β-ionone. These observations indicate that no obvious NADP^+^ channeling occurs within our fusion construct. The slightly higher conversion of β-ionone in the presence of LB-ADH may be due to higher NADPH concentrations produced by the combination of ADH and FDH.

## Discussion

Inspired by natural biosynthetic pathways, researchers have established a number of successful biocatalytic processes in which they combined multiple enzymes that catalyze biotic and abiotic sequential reactions in one pot^3^. Enzymes belonging to the same multienzyme cascade can be fused by cloning their genes together without a stop codon between them. An obvious advantage provided by this approach is that fused enzymes are produced as a single polypeptide. Most examples in the literature are dedicated to the construction of bifunctional units which have been established with representatives from different enzyme classes and recently reviewed^3,52,53^. In many cases, enzyme fusion positively affected enzyme properties compared to the unfused counterparts^9,13,23,26,54^. Nevertheless, fused constructs with significantly decreased activity have also been reported. For instance, loss of ADH activity in fusions between an ADH and a cyclohexanone monooxygenase (CHMO) as well as triple fusions between an ADH, a CHMO and an enoate reductase in particular enzyme order highlighted the importance of structural elements located near the termini which arguably contribute to proper enzyme folding, oligomerization and cofactor coordination^9,10^. Thus, mere prediction of a positive outcome for enzyme fusion remains problematic due to unique enzyme structures, and further research of different fusion enzyme constellations remains intriguing.

In this study we created a set of fusions between P450 BM3 4m and FDH2 for self-sufficient cofactor regeneration. Formate dehydrogenase was chosen as a fusion partner due to its high stability and the almost irreversible nature of the catalyzed formate oxidation. Our results demonstrate that expression levels were affected by the order of the fused enzymes and the fusion with BM3 4m at the N-terminal part, was produced as soluble protein in *E. coli* at a threefold higher level than all fusions with N-terminally located FDH2 component. This might be explained by the high solubility of P450 BM3 and/or a higher translation efficiency and folding when the gene sequence coding for P450 BM3 is localized at the 5′-terminus of the mRNA^55^.

The constructed fusions allow for simplified handling and exhibited an up to threefold increased P450 activity compared to the individual enzyme under the same conditions. In an attempt to rationalize the enhanced activity of the fusions, we analyzed different factors that may be responsible for this positive effect. Intuitively, one can surmise that proximity of multiple active sites can cause the increase in conversion due to the effect of substrate/intermediate channeling^56^. In case of the fusions studied in this work, the BMR site for binding NADPH and the FDH site for binding NADP^+^ should be located in close proximity to each other. NADP^+^/NADPH could theoretically be cycled between the two enzymes with the rates faster than diffusion in the bulk solution. This would imply that NADP^+^ is excluded from the bulk solvent to a certain extent and thereby unavailable to the competitive LB-ADH reaction. Ideally, no 2-pentanol would be detected. However, in the used setup we were not able to demonstrate that NADP^+^ is channeled directly from the BMR binding site to the FDH binding site. On the one hand, the k_*cat*_ values measured with BM3 4m are probably far too slow and do not allow to measure NADP^+/^NADPH channeling. On the other hand, conclusive evidence for substrate channeling in artificial fusions of several enzymes have rarely been reported (i.e. Refs.^23,57^). Moreover, proximity alone is not enough to increase the rate of multistep conversion and many other factors play a role^21,56^. Thus, we searched for other factors that could explain higher substrate conversion by the fusions.

First, we assessed the role of the cofactor regeneration efficiency: While the enzyme order in fusions FDH-BM3 or BM3-FDH seems to have almost no effect on FDH stability (Fig. 3c) and catalytic efficiency at high formate concentrations (Table 3), the presence of a less flexible or a longer linker in the fusion constructs F2B P1 and F2B-G4 resulted in higher FDH activity (Fig. 2a). FDH consists of a cofactor binding domain in the middle of the polypeptide chain and an HCOO^–^ binding domain which harbors the N- and C-terminus of the polypeptide chain^58^. Presumably, a rigid proline linker and a long glycine linker facilitate the adoption of the conformations that are required for HCOO^−^ binding at the appropriate domain of FDH in these fusions. Additionally, we observed higher FDH activity with the BF2 enzyme order (Fig. 2a). An improvement of FDH activity upon enzyme fusion has been reported for several constructs with FDH fused at the C-terminus. Investigations on fusions between FDH from *C. boidinii* and leucine dehydrogenase (LeuDH) from *B. sphaericus*^36^ revealed a 1.5-fold increase in FDH activity which was attributed to an 3- to 12-fold increased catalytic efficiency^36^. Different to our results, the order in which FDH was fused to a partner enzyme had a dramatic influence on FDH activity in the fusions of FDH from *M. vaccae* and 3-ketoacyl-(acyl-carrier-protein) reductase (KR) from *Synechococcus *sp. PCC 7942. The authors reported that the KR-FDH orientation with C-terminal FDH resulted in complete loss of FDH activity which could only be partially restored through the insertion of long linkers^37^.

Although the FDH activity within fusions was slightly higher when longer flexible glycine linker or a rigid proline linker were used, our results generally indicate that there was no substantial difference in expression and general catalytic performance of the fusions with different linkers. These findings contrast with some observations that linker type and length can significantly influence catalytic performance of fusions. For instance, a glycine-rich linker (10 amino acid residues) between a beta-glucanase and a xylanase increased activity of the fusion considerably compared to fusions without a linker^15^. A glycine-rich (30 amino acids) flexible linker between the styrene oxygenase (StyA) and styrene reductase (StyB) was found to increase soluble expression of the fusion as compared to the constructs with short and rigid linkers^59,60^. Likewise, linkers consisting of the amino acids Pro/Ala/Ser (PAS) of variable length (20 to 60 amino acids) between ADH and an aminotransferase fusions impacted expression levels and activity^13^. Probably, distance and flexibility imposed by linkers are not crucial for the fusion between P450 BM3 and FDH in this work. However, we tested only two linker types with a length of up to 20 amino acids (fusion construct G4). Any effects with a different linker type or increasing length should be further investigated.

Regarding the P450 activity of the fusions, to the best of our knowledge, fusion constructs comprising P450 BM3 and another enzyme have been created only once before. Phosphite dehydrogenase (PTDH) was used as a fusion partner to facilitate cofactor regeneration^26^. The authors observed increased total turnover numbers for PTDH-BM3 fusions with P450 substrates such as omeprazole and rosiglitazone, although kinetic parameters with lauric acid as substrate for P450 BM3 and phosphite as substrate for dehydrogenase were not significantly different compared to the individual enzymes. In our study, we observed up to threefold increased conversion of lauric acid, myristic acid and β-ionone by the fusions. Similar to the positive effects of enzyme order on FDH activity, we observed increased P450 activity (Fig. 2b). These observations were confirmed through the increased k_*cat*_ values for both the P450 component and the FDH component measured with both BF2 and F2B fusions. Some variations in K_M_^12-*p*NCA^ between the individual BM3 4m and the fusions F2B and BF2 might indicate some changes in the heme domain BMP. Furthermore, lower solvent stability of the fusions arguably stems from structural changes in the BMP active site of the fusion constructs that facilitate organic solvent access to the active site^61,62^. However, the differences in K_M_ values are not as pronounced as the difference between the k_*cat*_ values, particularly between the individual BM3 4m and F2B (Tables 3, 4). Furthermore, we recorded only minor changes in product distribution and coupling efficiency between fusion constructs and the individual BM3 4m. Thus, if alterations in the BMP heme domain occurred, they seem to not be the main factor responsible for the increased activities of the fusions.

Instead, the reduction rates measured with the artificial electron donors, ferricyanide and cytochrome c reduction, demonstrate that the reductase domain of BM3 4m (BMR) was significantly affected upon fusion. The F2B fusion can transfer electrons 1.5–3 times faster than BF2 and individual BM3 4m, which is likely responsible for higher activities of the fusions and can explain why F2B fusion is more active than BF2. A similar increase in the FAD reduction rate was determined in the experiments with P450 BM3 wild-type at very low enzyme concentrations (< 5 nM) which presumably led to dissociation of the active dimer into monomers with a less hindered access to FAD for NADPH^46^. Similarly, enzyme fusion might lead to conformational changes in the FAD-binding site, which in turn leads to an easier access for NADPH. Such structural changes could also alter FAD binding leading to decreased thermal stability of BMR within the fusion observed in this study.

Recently, the dimeric character of BM3 was evaluated by cryo-electron microscopy and two possible conformations have been postulated which are mediated by a flexible BMP heme domain^63^. While dimerization occurs at the BMR domains, BMP was found to be either distanced (inactive form) or proximal to BMR (active form). FDH naturally dimerizes in solution as well and in the fusion enzymes, it could arguably impose constraints on the mobility of the BMP domains and thereby result in the altered stability and activity of fused BM3 4m observed in our study. On the other hand, a spatially close arrangement of FDH and BM3 in the fusions could promote dimerization of one of the fusion partners with a higher dimer dissociation constant. Such an observation was reported with P450 BM3-PTDH fusions, in which a lower dissociation constant was measured for fused P450 BM3 compared to the individual enzyme^26^. Future investigations with structure modeling and MD simulations could help to shed more light on oligomerization and flexibility of the constructed fusions.

In conclusion, enzyme fusion between P450 BM3 and FDH resulted in constructs that were superior to the individual enzymes in terms of activity and k_*cat*_ values depending on the enzyme order in the fusions. These improvements can be attributed to an increased BMR activity with artificial substrates. The lower thermal and solvent stability of the fusion constructs indicate that structural alterations of the individual enzymes may occur in the fusion constructs that result in the observed improvements of catalytic properties.

## Methods

### Construction of fusions

Fusion of the genes coding for P450 BM3 (A74G/F87V/L188G/R471C) and formate dehydrogenase (D221Q) was conducted by overlap extension PCR using pET-28a(+)-*p450 BM3 4m* and pET-28a(+)-*fdh* as templates. Details on the thermal profiles for PCR and the cloning procedure are described in the Supplementary Information. The gene fragments were purified by gel electrophoresis and cloned into the pET-28a(+) vector by restriction and ligation using the *NcoI* and *EcoRI* restriction sites. Subsequently, *E. coli* DH5α were transformed with the constructs for propagation of the DNA material.

### Enzyme expression, purification, and quantification

A detailed description of the procedures on expression and purification is listed in the Supplementary Information. Briefly, expression of the fusion enzymes was conducted in *E. coli* BL21 (DE3) using Terrific Broth (TB) supplemented with kanamycin (30 µg/mL), FeSO_4_ (0.1 mM) and 5-aminolevulinic acid (80 µg/mL). After cultivation, the bacterial cells were centrifuged at 3000×*g* and 4 °C for 30 min and resuspended in 3 mL phosphate buffer (KPi, 50 mM pH 7.5, NaCl 300 mM, 0.1 mM PMSF) per gram cell wet weight. Sonication (Branson sonifier, BRANSON Ultrasonics Corporation) was utilized to disrupt the cells. After removal of cell debris (18,000×*g* and 4 °C for 30 min), fusion constructs and enzymes were purified by IMAC (5 mL HisTrap Crude FF, GE Healthcare) and subsequently by SEC (Superdex 200 Increase 10/300 GL). Enzyme quantification by CO-difference spectrum assay was conducted as described previously^64^. Quantification of FDH concentration, LB-ADH concentration and total protein concentration was conducted with Coomassie Brilliant Blue G-250 (RotiQuant, Roth) according to the manufacturer’s manual with a linear calibration curve based on bovine serum albumin (BSA)^65^.

### Determination of P450 and FDH activities

Volumetric activity of individual and fused BM3 4m was assessed with the 12-*p*NCA assay with NADPH as cofactor, while volumetric FDH activity was determined through the turnover of NADP^+^ and formate to NADPH and CO_2_^66^. Reactions for the 12-*p*NCA assay were conducted with purified enzymes (50 nM) in KPi (50 mM, pH 8.1) with 12-*p*NCA (0.2 mM dissolved in DMSO) and NADPH (1 mM). For the determination of FDH activity, samples consisted of KPi (50 mM, pH 7.5) with sodium formate (0.6 mM), NADP^+^ (0.5 mM), and at enzyme concentration of 100 nM. Reactions were initiated by the addition of NADPH for 12-*p*NCA turnover, and the resulting absorbance increase was recorded photometrically at 410 nm. Volumetric activities were calculated with ε_410_ = 13.2 mM^−1^ cm^−1^ for 12-*p*NCA. The generation of NADPH by FDH was measured at 340 nm after reactions were initiated with the addition of NADP^+^. Volumetric activities were calculated using ε_340_ = 6.22 mM^−1^ cm^−1^ for NADPH. Fold activity of the fusions was calculated relative to the volumetric activity of the individual enzymes.

### Measurements of BMR activity

The activity of BMR was determined through the reduction of potassium ferricyanide (PFC) and cytochrome c (from bovine heart, Sigma). Samples consisted of Tris–HCl (50 mM, pH 7.5), NADPH (0.3 mM) and either 2.5 nM enzyme with cytochrome c (0.05 mM) or 10 nM enzyme with PFC (1 mM). Reduction of substrate was measured at 420 nm for PFC (ε_420_ = 1.02 mM^−1^ cm^−1^) or 550 nm for cytochrome c (ε_550_ = 18.6 mM^−1^ cm^−1^)^67,68^. The absorbance decrease for reactions with PFC or cytochrome c with NADPH only was subtracted from the values of reactions that contained enzymes. Substrate turnover was calculated relative to enzyme concentration.

### Substrate conversion and determination of coupling efficiency

Substrate conversion was conducted at 25 °C for 10 min at an enzyme concentration of 50 nM. All substrates were dissolved in DMSO which resulted in 2% (v/v) DMSO in all samples. For the conversion of lauric acid and myristic acid, reaction samples contained either 0.5 mL or 1 mL KPi buffer (50 mM, pH 7.5) with substrate (0.2 mM), NADP^+^ (0.63 mM) and sodium formate (1.5 M). After incubation, internal standard (for lauric acid—undecanoic acid, for myristic acid—tridecanoic acid) was added to a final concentration of 0.1 mM. The reaction was quenched with the addition of 15 µL 37% HCl, extracted twice with 700 µL diethyl ether (1% HCl) and dried with anhydrous MgSO_4_. After solvent evaporation, reaction products were resuspended in 50–100 µL N,O-bis(trimethylsilyl)trifluoroacetamide containing 1% trimethylchlorosilane, derivatized at 80 °C for 30 min and analyzed by GC/MS.

Conversion of β-ionone was conducted in 0.2 mL KPi (50 mM, pH 7.5) with substrate (0.2 mM), NADP^+^ (1 mM) and sodium formate (1.5 M). Following the addition of either 1-decanol (0.1 mM) or 2-nonanol (0.1 mM) as internal standards, the samples were extracted with 0.2 mL ethyl acetate and the organic phase was analyzed by GC/MS. For the determination of coupling efficiency, reaction samples consisted of KPi (50 mM, pH 7.5, 1 mL) with substrate (0.2 mM), NADPH (0.1 mM) and enzyme (100 nM). Samples were incubated for 30 min at 25 °C and processed for GC/MS analysis as described above. Conversion of 0.1 mM substrate would equal a coupling efficiency of 100%.

### Kinetic measurements

Reactions with FDH (300 nM) were carried out in KPi buffer (50 mM, pH 7.5) and 2% v/v DMSO. Varying concentrations of NADP^+^ (0.05–1 mM) were reduced at a fixed concentration of sodium formate (1.5 M). Oxidation of sodium formate (10–600 mM) was measured at a fixed NADP^+^ concentration (2 mM). Reaction rates were quantified through the absorbance increase at 340 nm with ε_340_ = 6.22 mM^−1^ cm^−1^ for NADPH. Kinetic parameters for the enzyme-mediated (50 nM F2B, 100 nM BM3, 150 nM BF2, purified by IMAC) 12-*p*NCA conversion were determined in KPi buffer (50 mM, pH 8.1) and 2% v/v DMSO. Absorbance increase of 12-*p*NCA conversion was measured at 410 nm with varying concentrations of 12-*p*NCA (0.005–0.3 mM) and at a fixed concentration of NADPH (1 mM). Reaction rates were calculated with ε_410_ = 12-*p*NCA 13.2 mM^−1^ cm^−1^. The reaction rates were plotted against the varying substrate concentration and a *Michaelis–Menten* fit was applied with the software *Origin 9* to calculate V_max_, K_M_ and subsequently k_*cat*_.

### Product analysis by GC/MS

A GC/MS-QP2010 Plus system (Shimadzu, Tokyo, Japan) with a FS-Supreme-5 column (30 m × 0.25 mm × 0.25 µm Chromatographie Service GmbH, Langerwehe, Germany) was utilized for sample analysis. Description of the temperature profiles for the analysis of reactions with lauric acid, myristic acid, 2-pentanol and β-ionone are listed in the Supplementary Information (Supplementary Table S3). For the quantification of conversion and determination of coupling efficiency, substrate peak areas were normalized with the peak area of the respective internal standard. Substrate depletion was calculated by relating substrate peak areas of reaction samples to samples that contained no enzyme. Conversions of lauric acid, myristic acid, β-ionone and 2-pentanol were quantified using linear calibration curves with rising substrate concentrations and addition of the corresponding internal standards. Coupling efficiency for β-ionone was quantified with a linear calibration curve without the addition of an internal standard.

### Determination of thermal stability and melting temperature T_M_

To determine the thermal stability, enzymes were incubated at 25 °C, 40 °C and 45 °C for 10 min, cooled down on ice for 2 min and incubated at 25 °C for further 5 min. Subsequently, activitiy of BMP (50 nM) was measured with 12-*p*NCA (0.07 mM), NADPH (0.5 mM) in KPi (50 mM pH 8.1). BMR (5 mM) activity was measured by PFC (1 mM) reduction with NADPH (0.5 mM) in Tris–HCl (50 mM pH 7.5) and FDH activity was measured with formate (0.6 M) and NADP^+^ (1 mM) reduction in KPi (50 mM pH 7.5). The resulting values were normalized to the values measured at 25 °C.

The determination of the melting temperature was based on the *Thermo*FAD assay^48^. Samples consisted of enzymes (1.5 mg mL^−1^, purified by IMAC) in 20 µL KPi buffer (50 mM, pH 7.5) and were subjected to gradually increasing temperature from 15 to 95 °C in 0.5 °C increments (qPCR cycler qTOWER^3^ Analytik Jena, Jena, Germany). The SYBR green fluorescence filter was utilized to monitor FAD fluorescence at 520 nm after excitation at 470 nm. The maximum after derivation of the melting curve (software OriginPro 9.0) represents the T_M_ value.

### Determination of solvent stability

Solvent stability of enzymes was tested with the conversion of 12-*p*NCA (0.07 mM) in the presence of DMSO, ethanol, isopropyl alcohol and acetonitrile as described above. Substrate was dissolved in each solvent to concentrations that equal a final concentration of 0.07 mM 12-*p*NCA after the addition of 2% (3.6 mM 12-*p*NCA), 5% (1.4 mM 12-*p*NCA) and 10% (0.7 mM 12-*p*NCA) substrate solution to the sample. Activities measured in the reactions at 5% and 10% of solvent content were shown in relation to the values measured with 2% solvent content.

### Competitive reactions with LB-ADH

The *R*-specific alcohol dehydrogenase from *L. brevis* (LB-ADH, GenBank AJ544275.1) was heterologously expressed and purified as described elsewhere by anion exchange chromatography and subsequent hydrophobic interaction chromatography^69^. Conversion of β-ionone by the fusion enzyme F2B and 2-pentanol by LB-ADH was conducted in one pot. Reaction mixtures in KPi (50 mM, pH 7.5, 10 mM MgCl_2_) contained β-ionone (0.4 mM), 2-pentanol (0.5 mM), sodium formate (0.6 M) and DMSO (2% v/v). For reactions with individual ADH (1.5 µM), NADP^+^ (0.2 mM) was utilized. Reactions with individual F2B (50 nM) as well as with F2B (50 nM) with ADH (1.5 µM) contained NADPH (0.2 mM). The reactions were incubated at 25 °C up to 6 min in 1 min steps starting from 3 min and internal standard 2-nonanol was added (0.5 mM). The samples were extracted with 0.5 mL ethyl acetate and the organic phase was analyzed by GC/MS.

## Supplementary Information

Supplementary Information.
