# Supplementary material for: Genetic fusion of P450 BM3 and formate dehydrogenase towards self-sufficient biocatalysts with enhanced activity
Source: Sci Rep. 2021 Nov 4;11:21706. doi: 10.1038/s41598-021-00957-5 (PMC8568981; doi:10.1038/s41598-021-00957-5)
Supplement: Supplementary file 1 — Supplementary Information. [file 41598_2021_957_MOESM1_ESM.pdf]

Supplementary Information

Genetic fusion of P450 BM3 and formate dehydrogenase towards self-sufficient biocatalysts with enhanced activity

Aresenij Kokorin[1], Pavel D. Parshin[2], Patrick J. Bakkes[1#], Anastasia A. Pometun[2,3], Vladimir I. Tishkov[2,3], Vlada B. Urlacher[1*]

[1]Institute of Biochemistry, Heinrich Heine University Düsseldorf, Universitätsstr. 1, 40225 Düsseldorf, Germany

[2]Department of Chemistry, Lomonosov Moscow State University, 119991 Moscow, Russian Federation

[3]Laboratory of Molecular Engineering, Federal Research Centre “Fundamentals of Biotechnology” RAS, 119071 Moscow, Russian Federation

[#] Present address: Institute of Bio- and Geosciences, IBG-1: Biotechnology, Forschungszentrum Jülich GmbH, 52425 Jülich, Germany

[*] Corresponding author:

Vlada B. Urlacher

E-Mail: vlada.urlacher@uni-duesseldorf.de

Methods

Generation of fusion sequences

The genetic fusion of the P450 BM3 4m and FDH2 was conducted by overlap extension PCR. Amplification of pET-28a(+)-*p450 BM3* and pET-28a(+)-*fdh* by PCR with the respective primers added an *NcoI* 5’-restriction site to the N-terminal enzyme sequence and an *EcoRI* 3’-restriction site to the C-terminal enzyme sequence (Table S1). Furthermore, the utilized primers added overhangs to the 3’-end of the N-terminal enzyme and 5’-end to the C-terminal enzyme that were complementary. These overhangs were designed to code for the linker sequence and were used as a priming point for the fusion and linear amplification by PCR (thermal cycler temperature profiles in Table S2). The fused genetic sequences were subsequently inserted into the pET-28a(+) plasmid through the flanking *NcoI* 5’- and *EcoRI* 3’-restriction sites.

Primer list for the amplification and fusion of P450 BM3 and FDH

Supplementary Table S1. Primer list for the amplification and fusion of P450 BM3 and FDH. Primers designated with only *fw* or *rev* in the name were used for the amplification of the single gene sequences and for the whole fusion in the final amplification step. Primers containing either an *L*, *G1*, *G4*, *P1* in the name were used for the overlap amplification of two gene sequences. Underlined sequences mark restriction sites (*NcoI*: CCATGG, *EcoRI*: GAATTC). Bold sequences mark the linker sequences for the glycine linker (TAGCGGTGGTGGTGGATCCG or AGCGGCGGTGGCGGATCC and iterative repeats thereof) and proline linker (GAGCCGCCGCCGCCTAAA). Sequencing primers were used to verify the correct gene sequence of the fusion enzymes.

Primers for PCR	
Name	Sequence 5´-3´
BF fw	CAAACAATCCATGGGTCACCACCACCACCACCATTCGGGTACAATTAAAGAAATGCCTCAGCCAAAAAC
BF rev	GGAGCTCGAATTCTCAGACCGCCTTCTTGAACTTGGCGGCCTCTTC
BF L fw	GTGGGCTGGGGC**TAGCGGTGGTGGTGGATCCG**CAAAGGTCCTGTGCGTTCTTTACG
BF L rev	GGACCTTTG**CGGATCCACCACCACCGC**TAGCCCCAGCCCACACGTCTTTTGCGTATCG
FB fw	CAAACAATCCATGGGTCACCACCACCACCACCATTCGGGTGCAAAGGTCCTGTGCGTTCTTTAC
FB rev	CGGAGCTCGAATTCTTACCCAGCCCACACGTCTTTTGCGTATCGGCCTTTTTC
FB L fw	GCGGTCGCTA**GCGGCGGTGGCGGATCC**ATGACAATTAAAGAAATGCCTCAGCCAAAAACG
FB L rev	GTCATG**GATCCGCCACCGCCGCTA**GCGACCGCCTTCTTGAACTTGGCGGCCTCTTC
FB G1 for	GCGGTCGCT**AGCGGCGGTGGCGGATCC**ACAATTAAAGAAATGCCTCAGCCAAAAACG
FB G1 rev	**GTGGATCCGCCACCGCCG**CTAGCGACCGCCTTCTTGAACTTGGCGGCCTCTTC
FB G4 for	GCGGTCGCT**AGCGGCGGTGGCGGATCCGGCGGTGGCGGATCCGGCGGTGGCGGATCCGGCGGTGGCGGATCC**ACAATTAAAGAAATGCCTCAGCCAAAAACG
FB G4 rev	**GTGGATCCGCCACCGCCGGATCCGCCACCGCCGGATCCGCCACCGCCGGATCCGCCACCGCCG**CTAGCGACCGCCTTCTTGAACTTGGCGGCCTCTTC
FB P1 for	GCGGTC**GAGCCGCCGCCGCCTAAA**ACAATTAAAGAAATGCCTCAGCCAAAAACG
FB P1 rev	**TTTAGGCGGCGGCGGCTC**AGCGACCGCCTTCTTGAACTTGGCGGCCTCTTC
FB P4 for	GCGGTC**GAACCGCCGCCGCCTAAACCGCCGCCGCCTGAACCGCCGCCGCCTAAACCGCCGCCGCCTGAA**ACAATTAAAGAAATGCCTCAGCCAAAAACG
FB P4 rev	**TTCAGGCGGCGGCGGTTTAGGCGGCGGCGGTTCAGGCGGCGGCGGTTTAGGCGGCGGCGGTTCAGC**GACCGCCTTCTTGAACTTGGCGGCCTCTTC
Name	Primers for sequencing
S-BM3-R1	GTCGGCGATGTTCCAGCCGCCCTTCC
S-BM3-R2	CGCTTTGGGCGTTTTCAAGCTCTTCTTG
S-BM3-R3	GGCTGTTGAAGTTCTTTGCTTGCTACG
S-BM3-R4	CGTTTCCAAACGGTTTAAACGCATGC
S-BM3-R5	CAATATGCTCATCTGCATTTAGACGCTC

PCR thermal profile for the overlap extension and final amplification of gene fusions between BM3 and FDH

Supplementary Table S2. The appropriate temperature [°C] and duration [s] are depicted for each step. The 1. PCR for the extension by complementary overhangs was conducted for 6 cycles. Primers (designated with solely fw or rev in their names) were added in the 2. PCR for amplification with 30 cycles of the fused genes.

	Temperature[°C]	Time [s]	Cycles
1. PCR for the overlap extension			
Initial denaturation	98	120	6 Cycles
Denaturation	98	10	
Annealing	69	30	
Elongation	72	90	
2. PCR for the amplification of the fused genes			
Initial denaturation			30 Cycles
Denaturation	98	10	
Annealing	72	10	
Elongation	72	90	
Final Elongation	72	600	

Expression, purification and quantification of fusions

Expression of the fusion enzymes was conducted in *E. coli* BL21 (DE3). A single colony was transferred to 5 mL Lysogeny Broth (LB) supplemented with kanamycin and incubated at 180 rpm shaking and 37°C for 18 h. Thereafter, 4 mL of the culture were used to inoculate 400 mL Terrific Broth (TB) in a 2 L Erlenmeyer flask supplemented with 30 µg/mL kanamycin (30 µg/mL). The culture was incubated at 180 rpm shaking and 37°C to OD_{600} of 0.6-0.8 and subsequently supplemented with $FeSO_4$ (0.1 Mm), 5-Aminolevulinic acid (80 µg/Ml) and IPTG (0.1 mM). For fusion enzyme expression, the cells were further cultivated at 140 rpm shaking and 25°C for 18 h. Expression of individual enzymes was conducted at 160 rpm shaking and 30°C for 18 h. After incubation, the cells were centrifuged at 3000 x g at 4°C for 30 min. The supernatant was discarded, and the cells resuspended in 3 mL phosphate buffer (KPi, 50 mM pH 7.5, NaCl 300 mM, 0.1 mM PMSF) per gram cell wet weight. Disruption was conducted on ice by four cycles of 1 min sonication (Branson sonifier, BRANSON Ultrasonics Corporation, output control: 4, duty cycle: 40%) with 45 s pauses between each cycle. Cell debris was centrifuged out of suspension at 18 000 x g and 4°C for 30 min. The supernatant was loaded onto a Ni-NTA column (5 mL HisTrap Crude FF, GE Healthcare) connected to an ÄKTAprime system (GE Healthcare) for immobilized metal affinity chromatography. After impurities were washed off the column with KPi buffer (50 mM pH 7.5, 500 mM NaCl) containing 40 mM imidazole, the protein of interest was eluted with KPi containing 250 mM imidazole. The protein solution was concentrated by ultrafiltration (Vivaspin Turbo 30 kDa MWCO, Sartorius) and loaded onto a Superdex 200 Increase 10/300 GL column (GE Healthcare) connected to an ÄKTApurifier system (GE Healthcare) for further purification by size-exclusion chromatography. Protein fractions were eluted with KPi (50 mM, pH 7.5, 150 mM NaCl, 5% glycerol) and stored at -20°C. Individual FDH was purified by IMAC only, concentrated by ultrafiltration and subjected to buffer exchange to KPi (50 mM, pH 7.5, 150 mM NaCl, 5% glycerol) by SEC with a PD-10 column (GE Healthcare).

Size-exclusion chromatography

The protein solution was applied through a 500 µL loop. Protein concentration did not exceed the maximum capacity of the column according to the manufacturer manual. The samples were run at a flow rate of 0.75 mL/min and fractions were collected at a volume of 1 mL.

Temperature profiles for substrate analysis via GC/MS

Injection temperature of 250 °C, interface temperature of 285 °C and 200°C ion source temperature were set for the measurement of all substrates. The total ion current (TIC) was detected in split mode with the appropriate split values displayed beneath the temperature profiles. Fatty acid analysis was conducted at an m/z range from 40-400 with 1 µL injected sample. For the compound β-ionone, the m/z range was set from 40-350 with 0.5 µL injected sample. Analysis of both 2-pentanol and β-ionone ranged from m/z 30-350 with 0.5 µL injected sample.

Supplementary Table S3. Temperature profiles for the product analysis of substrate conversion experiments by GC/MS.

Analysis of myristic acid (C14)

Rate	Final temperature	Hold time
-	180	2
8	300	0
-	300	5

Split 10

Analysis of lauric acid (C12)

Rate	Final temperature	Hold time
-	150	1
10	260	0
40	300	3

Split 10

Analysis of 2-pentanol and β-ionone

Rate	Final temperature	Hold time
-	35	5
30	240	0
60	300	0

Split 5

Analysis of β-ionone

Rate	Final temperature	Hold time
-	120	2
20	240	2
50	300	1

Split 5

Results

Analysis of separate and fused enzymes by SDS-PAGE

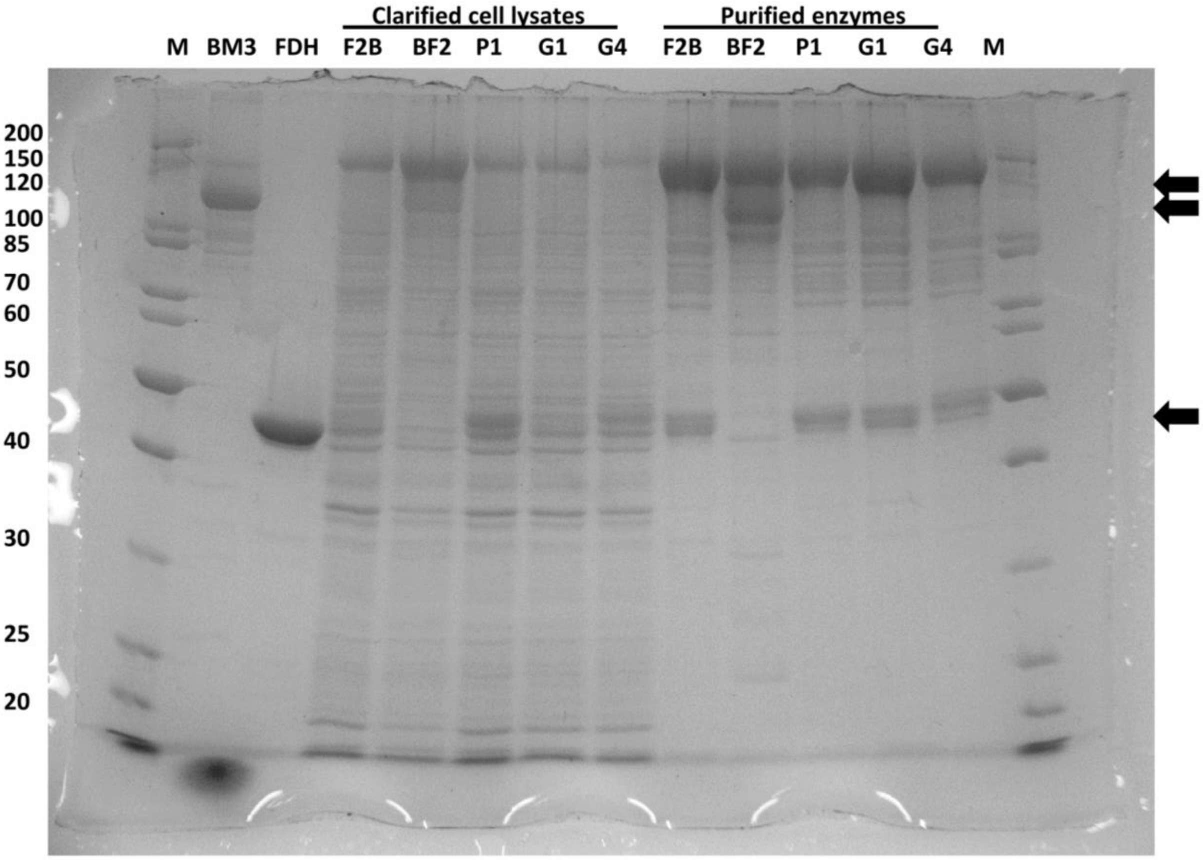

Supplementary Figure S1. SDS-gel (12%) of the clarified *E. coli* lysates and purified proteins. Arrows on the right side indicate the apparent molecular weights of the fusion proteins (150 kDa), BM3 4m (110 kDa) and FDH2 (45 kDa). Purified BM3 4m and FDH2 were used as references. For the lanes depicting cell lysates, approx. 10 µg of total protein were loaded. Lanes depicting purified proteins contained approx. 3 µg of total protein.

UV/Vis spectroscopy of individual BM3 4m and fusion constructs

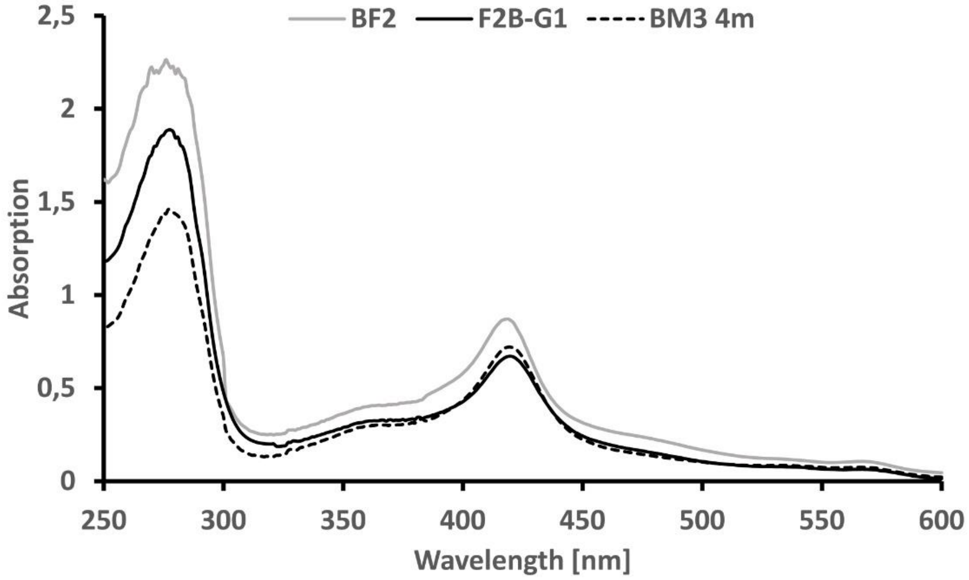

Supplementary Figure S2. UV/Vis spectra of individual BM3 4m (dashed line), BF2 (grey line) and F2B-G1 (black line). A protein concentration of 6 µM (determined by CO difference spectrum assay) was utilized for the measurements

Size-exclusion chromatography of the fusion enzymes F2B and BF2

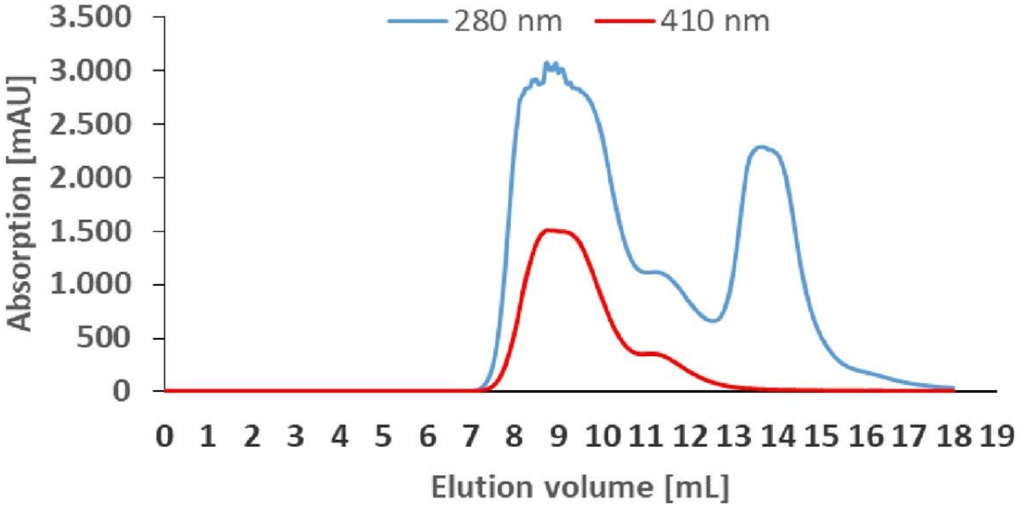

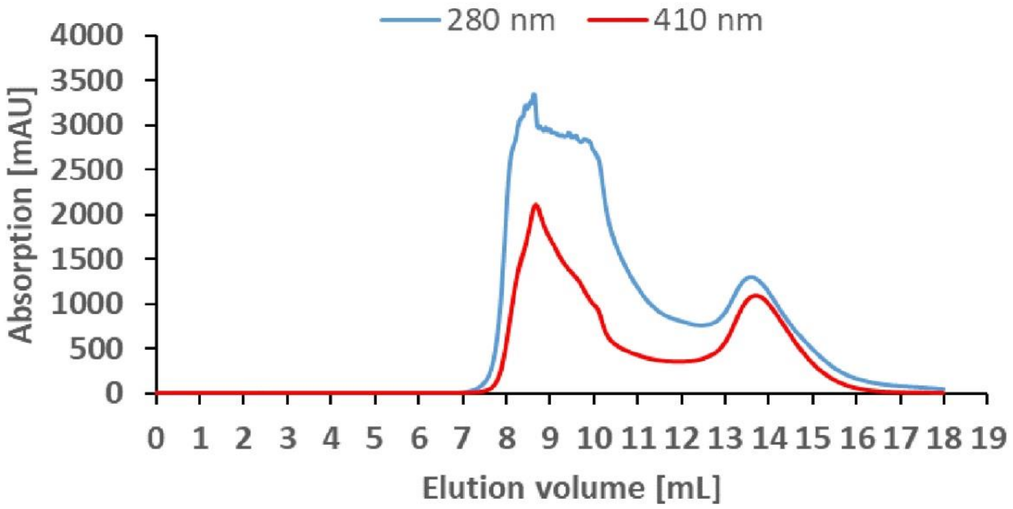

Supplementary Figure S3. Chromatograms of the fusion enzyme BF2 and F2B. The fusion enzymes elute with an elution volume of 8-10 mL. For the BF2 enzyme order, the additional peak at 13-15 ml can be attributed to cleaved-off monooxygenase domain of P450 BM3 with the absorption at 410 nm which is characteristic for the heme-group. The additional peak at 14 mL for the F2B order presumably stems from cleaved off FDH.

Product distribution of lauric acid conversion

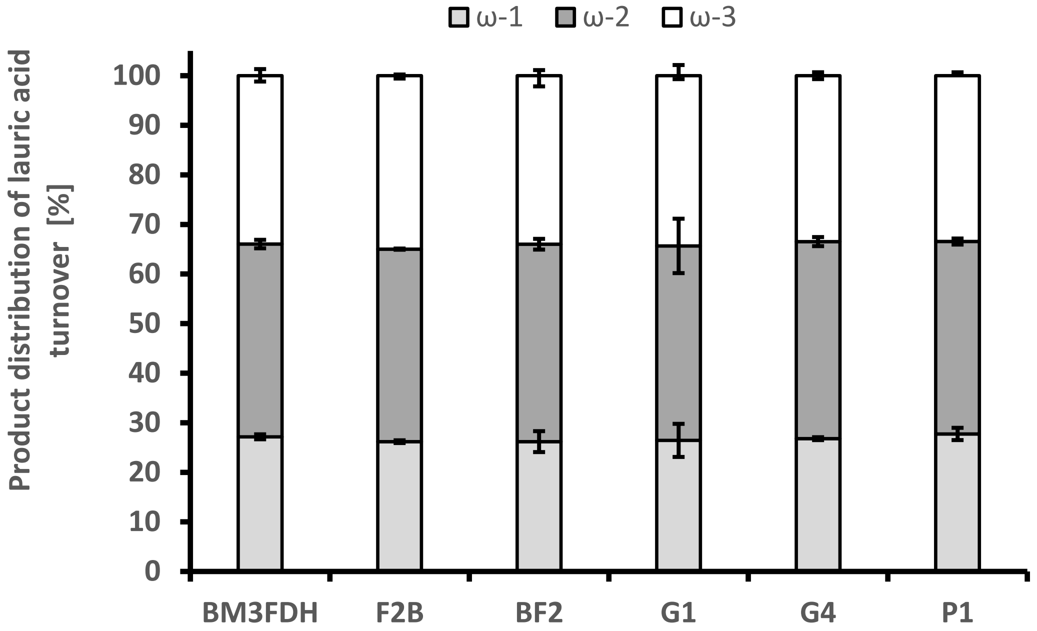

Supplementary Figure S4. Product distribution of lauric acid (C12) hydroxylation by BM3 and FDH and the fusion constructs F2B, BF2, F2B-G1, F2B-G4 and F2B-P1.

Product distribution of myristic acid conversion

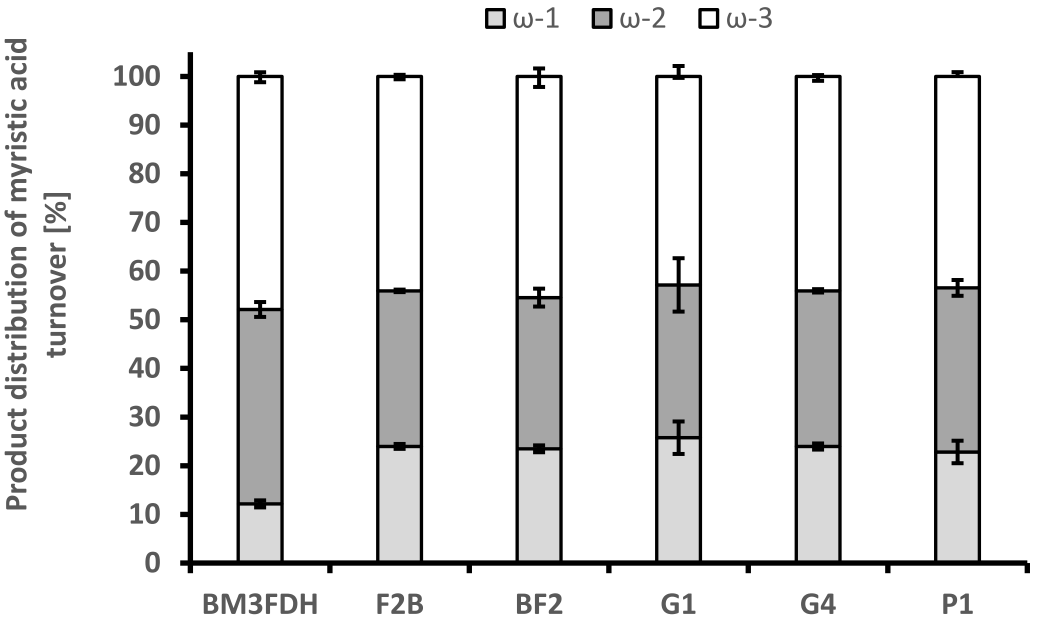

Supplementary Figure S5. Product distribution of myristic acid (C14) hydroxylation by BM3 and FDH and the fusion constructs F2B, BF2, F2B-G1, F2B-G4 and F2B-P1. .

Determination of kinetic parameters for F2B, BF2 and BM3 4m or FDH2

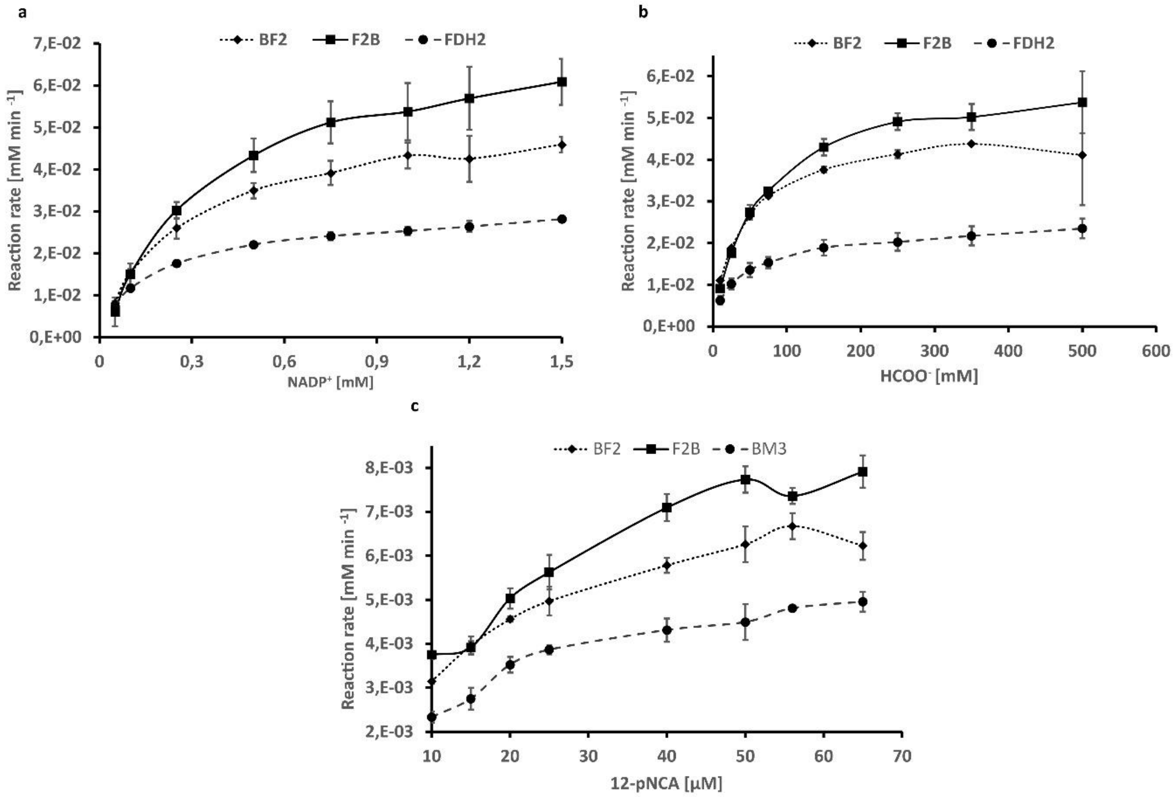

Supplementary Figure S6. Determination of kinetic parameters for F2B, BF2 and BM3 4m or FDH2. a) Reaction rate measurements for the determination of $HCOO^-$ oxidation with varying $NADP^+$ concentration. b) Reaction rate measurements for the determination of $NADP^+$ reduction with varying $HCOO^-$ oxidation. c) Reaction rate measurements at a constant NADPH concentration with varying 12-*p*NCA concentration. The reaction rates for the appropriate reactions were measured as described in the method section and plotted against the substrate concentration. The data was fitted to the *Michaelis-Menten* model with the software *Origin 9 Pro*. Data points were measured in triplicate and the standard deviation was calculated and displayed as the error bars.
